# P-341. Improving Outcomes for PWH with OUD: Five Years of Cohort Data from the UAB OBAT Clinic

**DOI:** 10.1093/ofid/ofaf695.559

**Published:** 2026-01-11

**Authors:** Leah J Leisch, Emma Kay, Ann Harshfield, Ellen Eaton

**Affiliations:** University of Alabama at Birmingham, Birmingham, Alabama; University of Alabama at Birmingham, Birmingham, Alabama; University of Alabama at Birmingham, Birmingham, Alabama; University of Alabama, Birmingham, Birmingham, Alabama

## Abstract

**Background:**

People with HIV (PWH) who experience opioid use disorder (OUD) face significant barriers to care, including poor treatment retention and comorbidities, like Hepatitis C (HCV). The objective of this study is to examine the comorbid conditions, substance use trends, and outcomes of patients attending an office-based addiction treatment (OBAT) clinic for PWH in Alabama.

**Methods:**

We evaluated PWH referred to the OBAT Clinic at the University of Alabama at Birmingham (UAB) HIV clinic from November 2019 to December 2024. We assessed baseline variables at clinic entry, such as HCV status and co-occurring stimulant use. We also evaluated OBAT clinic outcomes over the five-year period: retention on buprenorphine, HIV viral load suppression, hospitalizations, routine vaccination, and sexually transmitted infections (STI).

**Results:**

Of the 132 PWH who met the inclusion criteria of OBAT clinic referral, 102 attended the initial visit and were deemed appropriate for the clinic’s care. Among individuals with available data at baseline, 52/101 (51%) had an undetectable HIV viral load, 84/102 (82%) had co-occurring stimulant use, 36/96 (38%) reported symptoms of major depression (PHQ-9 > 10 and PHQ-2 > 3), and 29/102 (28%) had active Hepatitis C, as defined by detectable HCV RNA. Of those retained in OBAT care at 6, 12 and 24 months, 67/74 (91%) individuals were retained on buprenorphine at 6-months, 54/63 (86%) were retained on buprenorphine at 12-months, and 42/48 (88%) were retained on buprenorphine at 24-months. Further, 43/63 (68%) individuals were virally suppressed for HIV at 6-months, 38/64 (59%) were virally suppressed at 12-months, and 34/51 (67%) were virally suppressed at 24-months. 83/102 (81%) individuals received a routine vaccination (Hepatitis A, B and/or Influenza). Syphilis was the most common bacterial STI, with 20/102 (20%) of individuals receiving a diagnosis.

**Conclusion:**

In the first 5 years of operation, the UAB OBAT Clinic was able to provide comprehensive addiction and routine care to a vulnerable group with complex substance use and infections. This integrated clinic resulted in relatively high buprenorphine retention, viral suppression, and vaccination uptake, despite high rates of stimulant use, depression, and comorbid conditions at baseline.

**Disclosures:**

Ellen Eaton, MD, MPH, Gilead: Honoraria

